# Biopolymeric Inhalable Dry Powders for Pulmonary Drug Delivery

**DOI:** 10.3390/ph17121628

**Published:** 2024-12-04

**Authors:** Sara E. Maloney Norcross, Leanna P. K. Levin, Anthony J. Hickey, David B. Hill

**Affiliations:** 1 Technology Advancement and Commercialization, RTI International, Research Triangle Park, Durham, NC 27709, USA; 2 Joint Department of Biomedical Engineering, The University of North Carolina at Chapel Hill, Chapel Hill, NC 27599, USA; 3 Marsico Lung Institute, The University of North Carolina at Chapel Hill, Chapel Hill, NC 27599, USA; 4 Department of Physics and Astronomy, The University of North Carolina at Chapel Hill, Chapel Hill, NC 27599, USA

**Keywords:** dry powder inhalers, biopolymers, polysaccharides, poly(lactic-co-glycolic acid), polycaprolactone

## Abstract

Natural and synthetic biopolymers are gaining popularity in the development of inhaled drug formulations. Their highly tunable properties and ability to sustain drug release allow for the incorporation of attributes not achieved in dry powder inhaler formulations composed only of micronized drugs, standard excipients, and/or carriers. There are multiple physiological barriers to the penetration of inhaled drugs to the epithelial surface, such as the periciliary layer mucus mesh, pulmonary macrophages, and inflammation and mucus compositional changes resulting from respiratory diseases. Biopolymers may facilitate transport to the epithelial surface despite such barriers. A variety of categories of biopolymers have been assessed for their potential in inhaled drug formulations throughout the research literature, ranging from natural biopolymers (e.g., chitosan, alginate, hyaluronic acid) to those synthesized in a laboratory setting (e.g., polycaprolactone, poly(lactic-co-glycolic acid)) with varying structures and compositions. To date, no biopolymers have been approved as a commercial dry powder inhaler product. However, advances may be possible in the treatment of respiratory diseases and infections upon further investigation and evaluation. Herein, this review will provide a thorough foundation of reported research utilizing biopolymers in dry powder inhaler formulations. Furthermore, insight and considerations for the future development of dry powder formulations will be proposed.

## 1. Introduction

**Figure 1:**
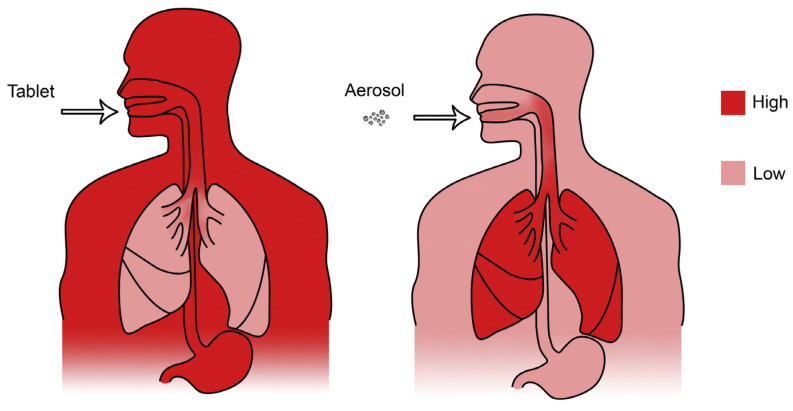
Drug exposure following oral (**left**) and pulmonary (**right**) delivery illustrating anticipated tissue concentration. Figure reproduced with permission [5]. Copyright 2016, Elsevier.

Pulmonary drug delivery has consistently garnered interest as an approach to achieve direct deposition, and therefore high concentrations, of drugs in the lungs (Figure 1). The lungs consist of a series of bifurcating airways beginning at the trachea and ending at the alveolar sacs [1]. The alveolar surface constitutes a large surface area, with estimates in the literature ranging from 70 to 140 m^2^ [2], representing a promising target for both localized and systemic drug delivery. Inhaled delivery of corticosteroids and bronchodilators has been frequently employed for lung diseases such as asthma and chronic obstructive pulmonary disorder (COPD). Inhaled delivery of such molecules allows for targeted delivery to the lungs and rapid onset of the intended effect [3]. The utility of inhaled therapy has expanded toward diseases where the lungs are a major site of infection, including tuberculosis, cystic fibrosis (CF)-related infections, and nontuberculous mycobacterial infections [4,5]. Pulmonary drug formulations including antibiotics, corticosteroids, bronchodilators, and biologics are constantly evolving to improve therapeutic utility, overcome emerging resistance, and increase patient access to treatments.

Several pharmaceutical aerosol products, which include a drug formulation and device, exist to deliver drugs to the lungs, including metered dose inhalers (MDIs), nebulizers, soft mist inhalers, and dry powder inhalers (DPIs) [6,7]. Following the global phase-out of chlorofluorocarbon propellants in medical products, many researchers have focused on the latter products [8]. Dry powder inhaler products offer many advantages over MDIs, nebulizers, and soft mist inhalers in that they utilize propellant-free dispersal mechanisms, solid-state drug formulations, and portable devices that do not require electricity [7]. In addition, DPIs are capable of delivering a range of doses (µg to mg range), making them a useful delivery mechanism for both low- and high-potency drugs. Herein, this review will focus specifically on formulations intended for DPI devices.

## 2. Dry Powder Inhalers

Dry powder inhaler technology has been available as a general therapy to relieve pulmonary disease for more than a century [9]. In the period since government agencies have regulated marketed drugs, the first dry powder inhalers appeared approximately 50 years ago [10]. The technology underpinning the delivery of dry particulate aerosols consists of the formulation of the powder, a metering system, and a device from which the powder is administered [10,11,12]. The earliest DPIs were intended to treat asthma [13]; however, the technology (e.g., devices, formulations) has since evolved as a treatment for COPD and infectious diseases [13,14].

It is generally accepted that particles with a 1- to 5-µm aerodynamic diameter are best suited to reach and deposit within the lungs [7]. Particles larger than 5 µm primarily deposit in the mouth, throat, and upper airways. While able to reach the alveolar region, particles of diameter less than 1 µm risk being exhaled [15]. As such, dry powder inhaler formulations vary in their composition to achieve this 1–5 µm ideal aerodynamic particle size. The predominant formulations include lactose as a carrier particle [16,17,18]. Jet-milled drug particles suitable for lung delivery (<5 µm) are generally difficult to disperse reproducibly. To aid in dispersion, they are commonly blended with large, coarse particles of lactose. Most asthma and COPD therapeutics employed in these formulations are potent pharmacological agents and may be used in as little as microgram quantities, and such small amounts are difficult to fill and administer from a metering system. Consequently, blending with lactose at low concentrations (<10%) allows efficient and reproducible filling and aerosolization of the therapeutic dose of drug particles. In contrast to the low-dose, high-potency drugs used in lactose blends, spray-dried particles may be constructed. These spray-dried particles exhibit low forces of interaction and allow high-dose delivery of low-potency drugs [19,20]. The delivery of the antibiotic tobramycin is a strong example of the application of this formulation strategy [21]. Spray-dried formulations may require stabilizing agents, such as buffer salts or sugars, and dispersion aids, such as amino acids. Notably, leucine has been used to change the surface composition of particles and facilitate dispersion by reducing the forces of interaction [22]. Spray drying and other methods of producing DPI formulations have created an opening to explore new excipients and drugs for pulmonary delivery.

The metering system employed to contain the nominal drug dose, preserve its stability, and facilitate its delivery varies between devices. The early inhalers were capsule-based systems, which continue to be a popular metering system [23]. In addition to acute relief applications, devices are increasingly used for maintenance therapy for diseases such as asthma and COPD. Thus, multi-dose systems were developed, including blister strips and blister disks. Systems containing multiple doses in a reservoir are also available [24]. Reservoir systems require an accurate sampling and dosing mechanism as part of the device that will allow both the removal of a dose and its delivery while preserving the integrity of the barriers to the reservoir, assuring stability of the drug and providing efficient and reproducible delivery through the life of the inhaler.

DPI devices have several important features that allow for the delivery of the drug aerosol [10,11,12]. Most devices employ dispersion on the inspiratory flow of the patient. Before the patient inhales through the device, a component must facilitate penetration of the barriers in the metering system to allow access to the dose. For each metering system, a different approach is required [23]. Capsule-based devices use pins that pierce the capsule, blister systems have a component that removes (i.e., peels away) the lidding foil from the blister, and reservoir systems have a mechanical dial that allows a sample to be extracted from the reservoir [23]. Each device then employs a designed path and geometry to achieve airflow to disperse the drug particles efficiently and reproducibly as the patient inhales through the device [25]. This flow path is usually defined in terms of resistance or pressure drop that controls the airflow, linear velocity, and shear that the powder experiences to effectively separate the respirable particles from the static powder bed into individual particles suitable for deposition in the lungs.

The combined DPI technology is considered a drug product and is evaluated in terms of the important performance characteristics of delivered dose and aerodynamic particle size distribution [26]. These properties define the quality of the product in terms that are relevant to lung deposition [27,28]. However, an interest in the prediction of lung deposition and disposition has increased greater attention toward in vitro methods of characterizing dry powder inhaler performance using physiologically relevant sampling and inspiratory flow conditions [7].

Overall, DPI technology is well understood and may be applied to the delivery of a wide range of therapeutic agents. While there are many considerations for the selection and development of a DPI, the first is likely to be the dose (i.e., low or high) requirement [29]. In addition to assessing dose requirements, physiological barriers to successful drug delivery must be considered when preparing the drug formulation.

## 3. Physiological Barriers to a Successful Inhalable Dry Powder

**Figure 2:**
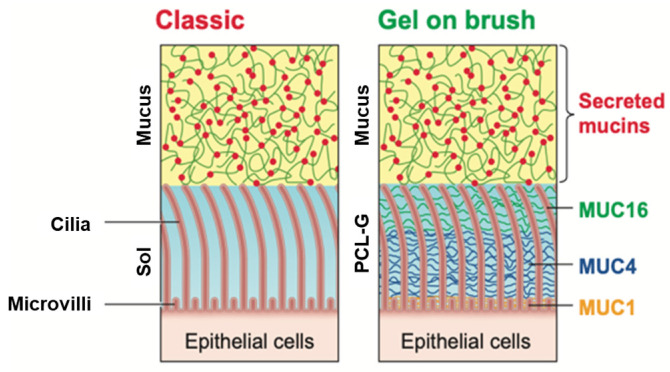
The airway surface layer. (**Left**): representation of the classic gel-on-liquid model showing a mucus layer (comprised of gel-forming mucins MUC5AC and MUC5B) and the periciliary layer (PCL) as a liquid-filled “sol” domain. (**Right**): gel-on-brush model depicting a MUC5B- and MUC5AC-populated mucus layer juxtaposed to the PCL comprised of “brush-like” epithelial-tethered mucins MUC1, MUC4, and MUC16. Figure reproduced with permission [32]. Copyright 2022, American Physiological Society.

To be successful, DPIs must overcome the innate protection offered to the respiratory tract by the airway surface layers (ASL). Our conceptual understanding of the ASL has evolved over the past decade [30,31,32]. Classically, the ASL was described as a two-phased system comprised of a viscoelastic mucus layer containing high molecular weight secreted mucin glycoproteins MUC5B and MUC5AC flowing atop the periciliary layer and glycocalyx (PCL-G), a simple aqueous layer in which cilia beat to clear mucus, i.e., “gel-on-sol” classic model (Figure 2). This view of the ASL captured the polymeric nature of MUC5B and MUC5AC interactions that give the mucus layer its characteristic concentration-dependent viscoelastic properties. From a polymeric point of view, the porosity, or mesh size, of mucus is defined by the concentration and size of the mucins. As the concentration of mucins increases, the mesh size of the layer decreases [32,33,34]. The gel-on-sol model, however, did not account for (a) why the secreted mucins did not interpenetrate the PCL-G under normal conditions nor (b) the collapse of the PCL-G in the presence of hyperconcentrated mucus, which is a hallmark of cystic fibrosis (CF) airway disease. As revealed by size exclusion experiments as well as osmotic pressure measurements of the ASL, the PCL-G is best described as a polymeric brush rather than a “sol” (Figure 2). The membrane-tethered mucins (TMs) MUC1, MUC4, and MUC16 coat the cilia and microvilli, producing a “gel-on-brush” architecture of the ASL, redefining the structure of the PCL-G [30]. One striking success of the “gel-on-brush” model is its ability to predict how the hyperconcentration of mucus that is associated with CF and other muco-obstructive pulmonary diseases osmotically draws water out of the PCL-G, collapsing cilia and slowing mucociliary clearance (MCC) [30].

**Figure 3:**
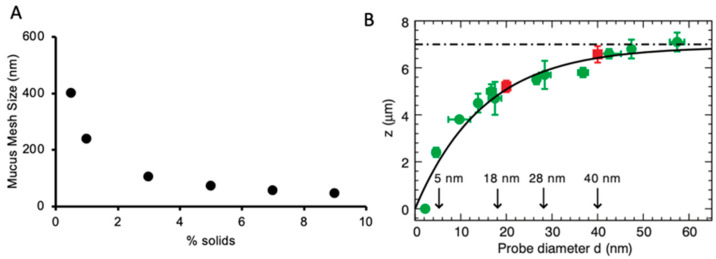
ASL mesh sizes. (**A**) Calculated mucus mesh size vs. mucus concentration. (**B**) The dependence of exclusion of the PCL-G (z) vs. the size of dextran molecules (green) and polystyrene particles (red). Figure reproduced with permission [30]. Copyright 2012, American Association for the Advancement of Science.

The polymeric nature of the PCL-G layer dictates that there is a concentration-dependent mesh size, or spacing between the mucins, that depends on the concentration and radius of gyration (R_g_) of these large-chain polymers. For mucins, the mesh size, or correlation length (*ξ*), scales as *ξ* ∝ [mucin]^(−3/4)^. As the concentration of mucus increases from normal levels of 1% organic solids (os) to a CF-like 7–10% os, the mesh size of the mucus layer decreases from 400 nm to <50 nm (Figure 3A) [30]. In addition to the restrictions of hyperconcentration on mucus mesh size, the increased osmotic modulus of hyperconcentrated mucus collapses the PCL-G, decreasing the size of probes capable of penetrating this layer from ~50 nm when the layer is fully extended to <5 nm when collapsed (Figure 3B) [30]. Together, the mucus and PCL-G layers have the ability to physically restrict the penetration of particles >50 nm under basal conditions, a size that is similar to many used for inhaled gene therapy vector delivery [35]. In pathological conditions like CF, the barriers may restrict particle penetration to ~5 nm.

In addition to the complications of mucus hyperconcentration, the persistent infections and inflammation associated with CF airway disease alter the ASL in several ways that may impact the penetration of drugs and biologics to cellular surfaces. The recruitment of neutrophils to the ASL alters the polymeric composition of the mucus layer by the addition of DNA-containing neutrophil extracellular traps (NETs). Indeed, while the concentration of mucins is increased 5- to 10-fold in CF compared to normal airways, the concentration of DNA is increased over 100-fold (Table 1) [36,37]. It is hypothesized that as these DNA NETs spread through and across the mucus layer, they will inter-penetrate and interact with the mucin network. DNA will be present in both the mucus bulk and skin layers. The cellular debris associated with NETs may facilitate mucin–mucin and mucin–DNA interactions as well as chemically trap drugs and biologics. Finally, the byproducts of inflammation increase the expression and concentration of tethered mucins in the PCL-G [38], potentially decreasing the mesh size of this layer.

The pathological changes in the ASL consequent to CF disease indicate that drugs and biologics may need to be co-administered with agents that make the mucus and/or PCL-G more penetrable. Inhaled hypertonic saline (HS) is widely used to treat CF and will ideally decrease the concentration of pulmonary mucus by drawing fluid from the underlying epithelium into the ASL, increasing its mesh size and penetrability to drugs and biologics. Further, restoring proper hydration of the mucus layer will ensure that the PCL-G can occupy its full 7 μm height, producing a maximal PCL-G mesh size. Notably, HS has the potential to disrupt the mucus skin layer by temporally dis-adsorbing the hydrophobic mucin domains from the air interface by adding liquid to the skin layer, thereby potentially making the mucus layer easier for drugs and biologics to penetrate. A complementary therapeutic strategy is to degrade the polymeric components of the mucus layer, mucins, and DNA. As mucins are stitched together from monomers into long-chain polymers by disulfide bound, agents such as tris(2-carboxyethyl)phosphine (TCEP), N-acetyl cystine (NAC, or Mucomyst), and dithiothreitol (DTT) have been shown to decrease the molecular weight of mucins and improve the rheological properties of mucus [41,42,43,44]. Likewise, DNA-cleaving agents such as dornase alfa (Pulmozyme) have been shown to decrease the pathological mucus viscosity and elastic modulus [45] by degrading the molecular weight of DNA [46]. In addition to reducing the viscoelastic properties of mucus, these reagents increase the mesh size of mucus by decreasing the molecular weight (and size) of the polymeric species [44]. Finally, the use of surfactants may decrease mucin-mucin, mucin-DNA, and mucin-protein interactions which stiffen mucus and effectively increase the molecular weight of the polymers present in mucus. Surfactants have been shown to decrease the surface tension of mucus as well as its viscosity [31]. Like HS, inhaled surfactants may be able to disrupt the mucus-skin layer and increase the layer’s penetrability to inhaled drugs and biologics.

The chemical and physical trapping of the mucus layer may hinder the effective delivery of inhaled therapeutics, especially when muco-obstructive pulmonary diseases are further limiting the mucus mesh size. As a result, many researchers have begun to evaluate DPI formulations that incorporate biopolymers to improve pulmonary delivery through varying mechanisms. A few of these mechanisms include incorporating mucolytic biopolymers into dry powder microparticles (MPs) to enhance permeation through the mucus layer and utilizing biopolymers that extend the residence time of the drug in the lungs to aid in drug diffusion to the target site upon particle dissolution.

An additional consideration that must be assessed in the formation of a successful DPI is the generation of the airway that is being targeted. Muco-obstructive pulmonary diseases such as CF [47], COPD [48], and asthma [49] are typically expressed in the distal lung, which is home to small airways (<2 mm in diameter) that constitute a large surface area of the lung. Often, the small airways in patients with these pulmonary diseases are obstructed by mucus plugging [50], which would prevent inhaled DPIs from reaching the distal lung. Structurally, the small airways are denoted by a lack of submucosal glands [32], an increase in the surfactant concentration [51], and a decrease in the percentage of ciliated cells lining the epithelium [52]. Whether the decreased ciliation in the small airways and a reliance on surfactants to help drive clearance make these areas of the lung more susceptible to the deleterious effects of mucus hyperconcentration, such as decreases in mucociliary clearance and mucus plugging, is an area of active research. Importantly for this review, researchers should consider region differences in the ASL in terms of mucus concentration, composition, and degree of ciliation to optimize the formulation and downstream efficacy of DPIs.

Furthermore, an important challenge faced by all inhaled therapeutics is pulmonary macrophages. Macrophages are the most numerous immune cell types found in the lung, with their primary physiological function being the phagocytosis of foreign matter in the airways [53]. While macrophages beneficially remove cellular debris and excess surfactant, their ability to take up a wide range of matter deposited on the airways allows them the potential to sequester inhaled therapeutics before they can reach the cellular epithelium. Additionally, as macrophages can induce inflammation [54], their presence may cause additional fluid and mucins to be secreted into the airways, enhancing the challenge to inhaled therapeutics [55].

## 4. Biopolymeric Microparticle Structure

### 4.1. Types of Biopolymeric Microparticles

**Figure 4:**
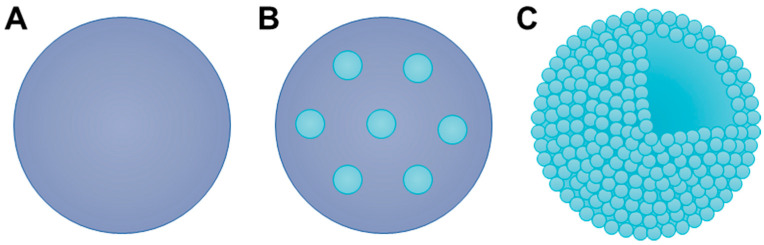
Biopolymeric MPs for dry powder systems, including (**A**) MPs, (**B**) nano-embedded MPs, and (**C**) porous NP aggregates.

For success as a DPI formulation, the majority of the powder must have an aerodynamic diameter between 1 and 5 µm. As described in Section 2, the composition of DPI formulations typically includes either micronized drugs combined with a coarse carrier molecule or engineered low-density drug-based MPs. When we discuss inhalable MPs, we often consider particles in the micron size with a homogenous composition (Figure 4A). However, additional categories of inhalable MPs exist, mainly nano-in-microparticles (Figure 4B,C) [56]. In these situations, nanoparticles (NPs) are pre-formed prior to undergoing a technique to prepare particles in the micron size range. To form nano-embedded MPs, polymeric NPs are formed and then incorporated throughout a hydrophilic matrix, such as lactose or mannitol, during preparation to form a micron-scale particle (Figure 4B) [56,57,58]. Alternatively, porous NP aggregate particles (PNAPs) are prepared when pre-formed polymeric NPs are dried into MP aggregates with or without inert excipients (Figure 4C) [59]. Protective excipients may be included during PNAP formation to prevent the destruction of the NPs [60]. Both nano-embedded NPs and PNAPs seek to utilize the benefits of the nano and micro scale [58,60]. Ideally, the micron-sized particles will allow for sufficient deposition in the lungs, where the NPs will disperse into their primary size. NPs may possess beneficial attributes for pulmonary delivery, such as providing sustained drug release, targeting specific locations for delivery, and penetrating through physiological barriers, such as the ASL [59,61].

### 4.2. Techniques for Preparing Biopolymeric Microparticles

Polymeric NPs and MPs, especially those prepared with poly(lactic-co-glycolic acid) (PLGA) are frequently prepared by forming an emulsion and then evaporating the organic solvent [56]. Double emulsions, where droplets of the dispersed phase have smaller dispersed droplets within them, can also be utilized for encapsulating hydrophilic materials that suffer from low encapsulation efficiency when prepared with a single emulsion method [62]. For charged polymers, ionotropic gelation can be employed to prepare nano- or microparticles. Briefly, the dropwise addition of a crosslinking agent into a polymer solution, or vice versa, will result in the formation of polymeric nano- or microparticles [56].

MPs and nano-in-microparticles are frequently fabricated using spray drying methods. For MP formulation, spray drying, freeze drying, or spray–freeze drying can be employed on a solution of polymer and drug to form uniform particles. These same drying techniques can be used with a suspension of NPs, such as those prepared using an emulsion and solvent evaporation process or ionotropic gelation. Spray drying is a fast, one-step, scalable process that transforms a solution or suspension into a dry particulate form through atomization and drying with a hot gas [56]. While frequently considered a superior method, spray drying does require the sample to experience increased temperature and shear forces, which may not be suitable for all sample types. Freeze drying is also commonly used, wherein the solution or suspension is frozen, and the solvent is removed via sublimation [63]. Depending on the precursor solution or suspension, the resulting product may or may not have a particulate nature. Spray–freeze drying is a three-step process where a solution or suspension is atomized, solidified through contact with a cold fluid, and dried through sublimation of the solvent [63]. Spray–freeze drying is not as widely available as spray drying or freeze drying but is often used for preparing dry powders of biologics. While these techniques encompass a majority of the prepared biopolymeric formulations, a subset of researchers use more specialized or unique methods for the preparation of dry powder MPs.

## 5. Natural and Natural-Derived Biopolymers in DPI Formulations

### 5.1. Natural Biopolymer Overview

**Figure 5:**
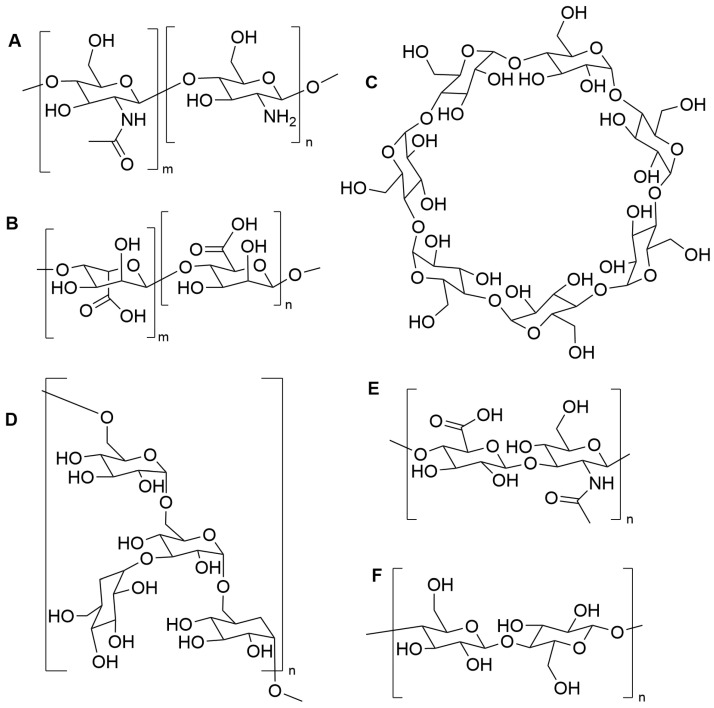
Chemical structures of natural polysaccharides, including (**A**) chitosan, (**B**) alginate, (**C**) β-cyclodextrin, (**D**) dextran, (**E**) hyaluronic acid, and (**F**) cellulose.

Naturally occurring biopolymers are an attractive option for DPIs as they are typically biocompatible, well-tolerated, and induce less inflammation than synthetically formulated molecules and polymers. Beyond the therapeutic effects of these polymers in their native form, they can be chemically augmented to serve as time-release carrier vehicles for therapeutically active molecules, as has been demonstrated for chitosan, alginate, and hyaluronic acid (Figure 5) [64,65,66,67,68]. Biopolymers such as gelatin, alginate, and others can be formulated into NPs that are designed to be deposited onto specific airways of the lung [69,70,71] and to overcome the innate barrier properties of the lung [70,71]. NPs and MPs are desirable for the delivery of therapies that are not well suited for direct inhalation, such as gene therapy vectors [70,71]. The development of nano- and microparticle systems of natural biopolymers and chemically modified natural biopolymers for DPI delivery has shown an increase in research interest. However, such systems have not yet been FDA-approved for inhalation.

### 5.2. Protein-Based Biopolymers in DPIs

Naturally occurring protein biopolymers, such as collagen and its partially hydrolyzed degradation product, gelatin, are frequently employed in drug delivery applications [72,73]. Notably, gelatin, a protein with 18 amino acids, has been described as a polymeric excipient in DPI formulations [74,75]. In 2004, Sham et al. prepared gelatin NPs of 242 ± 14 nm, which were then spray-dried in a solution containing large quantities of lactose [75]. After spray drying, the NPs increased to a geometric size of 320 ± 58 nm but exhibited a mass median aerodynamic diameter (MMAD) of around 3 µm, indicating that they could be suitable for inhaled delivery [75]. More recently, Behrend-Keim et al. prepared gelatin particles with model drugs, cromoglicate sodium and ipratropium bromide, through ionotropic gelation and spray drying [74]. For both drugs, the MMAD ranged from 3.4 to 4.5 µm and had a fine particle fraction (FPF) greater than 55% with respect to the emitted dose. Particle formulation slowed the release of the drug and also exhibited swelling behaviors, where the particle could reach 10 µm within minutes of exposure to solution. The authors postulate that this property could allow the particles to swell after deposition in the lungs, prolonging lung retention [74]. Of note, a myriad of proteins and peptides have been incorporated within polymeric DPI formulations as the active ingredient rather than as a polymeric excipient. These reports have been extensively reviewed by Marante et al. and are beyond the scope of this review [76].

### 5.3. Polysaccharides and Polysaccharide Derivatives in DPIs

#### 5.3.1. Chitosan

Chitosan (Figure 5A) is a naturally occurring linear biopolymer that has been used in a variety of applications. Primarily, chitosan has been modified in a number of ways to allow the inhaled delivery of therapeutic agents to the airways. Modified chitosan-based NPs have shown promise for the treatment of lung cancer [77] and type 2 diabetes [78]. Additionally, chitosan has been modified to store and release nitric oxide to eradicate *Pseudomonas aeruginosa* biofilms [65]. This material was also able to degrade both mucins and DNA, the two principle polymeric components of mucus whose hyperconcentration is associated with disease severity and increased mucus viscoelasticity [64]. Due to the prevalence of chitosan and its natural properties, it has been widely studied as an excipient in DPI formulations.

In 2006, Corrigan et al. prepared X-ray amorphous chitosan MPs containing salbutamol sulfate via spray drying [79]. The chitosan MPs released the drug rapidly, with >90% release in 5 min, and demonstrated a greater FPF than micronized salbutamol sulfate alone (28–36% versus ~1.5%) [79]. Similarly, Learoyd et al. fabricated two chitosan-based MP drug release systems [80,81]. Here, three molecular weights of chitosan were compared, with low molecular weight (LMW) chitosan at <190 kDa, medium molecular weight (MMW) chitosan between 190 and 310 kDa, and high molecular weight (HMW) chitosan at >310 kDa [80,81]. The MMAD for particles encapsulating terbutaline sulfate or beclomethasone dipropionate was 1.6–2.3 µm or 2.1–3.2 µm, respectively [80,81]. In the case of terbutaline sulfate, the FPF tended to decrease with increasing chitosan molecular weight, with LMW chitosan giving an FPF of 82.0 ± 3.1% and HMW chitosan giving an FPF of 55.8 ± 1.5% [81]. Notably, dissolution trended with the molecular weight of chitosan as well. Pure drug of either terbutaline sulfate or beclomethasone dipropionate dissolved in 5 or 20 min, respectively. For terbutaline sulfate or beclomethasone dipropionate, LMW, MMW, and HMW chitosan increased this to 30 min or 2 h, 60 min or 9 h, and 2 h or 12 h, respectively [80,81]. These studies demonstrate chitosan’s utility in extending the dissolution of drugs, which may allow for sustained lung retention.

In a study by Yang et al., chitosan (400 kDa) and chitosan oligosaccharides (5 kDa) were combined with leucine and the lipid dipalmitoylphosphatidylcholine (DPPC) at varying ratios to optimize the delivery of fanhuncaoin [82]. The top combination of 10/45/33.75/11.25/0.4 *w*/*w*/*w*/*w*/*w* of fanhuncaoin/leucine/chitosan/chitosan oligosaccharides/DPPC gave an MMAD of 2.24 µm and FPF of 51.29% [82]. In a later study by Ni et al., cinaciguat, a model hydrophobic drug, was prepared as nanocrystals using high-pressure homogenization and then encapsulated within chitosan MPs via spray drying [83]. The experimental MMAD was between 4 and 4.5 µm, and the FPF ranged from 40 to 45% [83]. Of note, the delivery was found to be dependent on inhalation efficiency, which could become problematic in treating patients with decreased lung function.

In a different approach for fabricating chitosan MPs, tripolyphosphate (TPP) has been used to form chitosan NPs through ionic gelation. Itraconazole was formed into chitosan– TPP-based NPs through ionic gelation, with NPs of approximately 190–240 nm size [84]. Spray drying the NPs with excipients, including lactose, mannitol, and leucine, led to the formation of inhalable MPs [84]. The FPF of the MPs formed without additional excipients was 16.3 ± 1.6%, but this metric could be increased to >30% when excipients were included [84]. Prothionamide, a second-line tuberculosis drug, was also prepared into chitosan/TPP NPs [85]. The particles were then lyophilized with mannitol as a cryoprotectant. The optimal formulation boasted an FPF of 81.19% with an MMAD of 1.76 µm (GSD = 1.96) [85]. The use of this formulation increased residency in the lung to more than 24 h [85]. The evaluation of freeze-dried chitosan/TPP NPs encapsulating rifampicin led to similar lung retention, where sustained release was observed up to 24 h in vitro and demonstrated extended residence time in the lungs of rats [86]. For the rifampicin particles, the MMAD was 3.3 ± 0.18 µm, and the FPF was 33.27 ± 0.87% [86].

Lastly, Arauzo et al. utilized a different approach to preparing chitosan particles [87]. Here, an electrospray technique was utilized to create chitosan particles loaded with ciprofloxacin at a sub-micron size (386.1 ± 248.5 nm with HMW chitosan and 501.1 ± 276.3 nm with LMW chitosan) [87]. The authors note that the high surface charge of the sub-micron particles, at +45 mV, gives the particles mucoadhesive properties [87]. However, neither in vitro nor in vivo aerosol testing was performed. As a result, the utility of this system for inhalation cannot yet be discerned.

#### 5.3.2. Alginate

Alginate (Figure 5B) is a linear carbohydrate polymer that has appeared in a variety of inhaled applications. It has been used as an inhaled formulation to reduce the viscoelastic properties of mucus and increase mucociliary clearance with limited effects on patients’ pulmonary function [88]. Inhaled alginate oligosaccharides have been shown to be well tolerated by patients in clinical trials, where the polymer decreases sputum viscoelasticity and may decrease the severity of *Burkholderia cepacia* infections in CF [88,89]. Beyond these studies of direct inhalation of alginate, alginate has been modified to donate nitric oxide to both eradicate CF-relevant bacterial biofilms and reduce the viscoelastic properties of mucus [68,90].

Ceschan et al. fabricated MPs of atenolol and alginate at varying ratios using spray drying, with drug loading from 0.30 to 0.63 g drug/g solid [91]. The theoretical MMAD was calculated to be in the range of 4.97 to 8.87 µm, but experimental aerodynamic testing was not performed [91]. A model protein, insulin, was formulated as a dry powder with a range of excipients [92]. The top formulation included mannitol, alginate, and sodium citrate excipients, exhibiting an MMAD of 2.51 ± 0.02 µm (GSD = 1.77 ± 0.02) and FPF of 81.13 ± 0.57% [92]. The formulation remained amorphous after 6 months of storage [92].

Alginate MPs (geometric size of 4.45 ± 1.40 µm) were prepared by Arauzo et al. in 2022 via an atomization process [93]. Briefly, the alginate solution was atomized into droplets, which were collected into a solution of barium chloride. The particles were then washed, collected, and dried. Lastly, the particles were mixed in a drug solution of colistin sulfate for drug loading [93]. Unexpectedly, the colistin sulfate was located on the surface of the particles with no drug inside the particles, and the drug was released in its entirety within 4 h [93]. The hollow nature of the particles may allow for an appropriate aerodynamic profile, but such testing was not conducted in this report.

Duong et al. recently reported on the preparation of alginate aerogel MPs as a system to deliver beclomethasone dipropionate [94,95]. The alginate aerogels were prepared using a gelation-emulsification method, and subsequently, the drug was loaded using supercritical fluid-assisted impregnation. Drug loading was found to be approximately 6–7 wt% [94,95]. The FPF from two reports was found to be 68–72% [95] and 78–80% [94]. In vitro and ex vivo studies were conducted with the alginate aerogel particles and demonstrated success with reasonable drug-to-excipient ratios [94].

#### 5.3.3. Cyclodextrin

Cyclodextrin (CD; Figure 5C) is a cyclic oligosaccharide with a hydrophobic inner cavity and hydrophilic exterior [96]. Cyclodextrin, in its 6-(α), 7-(β), and 8-(γ) membered ring form, has been used for a variety of drug delivery applications, especially those that require solubilization of the drug. There are also multiple commercially available variants of cyclodextrin, such as the popular hydroxypropyl-β-cyclodextrin (HP-β-CD) modification, which improves the water solubility of native β-cyclodextrin [97]. Cyclodextrins have shown increasing prevalence in studies preparing DPI formulations.

Dufour et al. demonstrated an improvement in respirable fraction and a reduction in permeability through a layer a Calu-3 cells as a result of spray drying budesonide with HP-β-CD [98]. The FPF of budesonide with a lactose carrier was 26.24 ± 3.46%, but the spray-dried complex increased this metric to 44.05 ± 1.65% [98]. The authors describe that the reduction in permeability across Calu-3 cells may translate to reduced permeability across the lung epithelium, improving lung retention of the drug [98]. Suzuki et al. fabricated a spray-dried co-formulation of HP-β-CD with two drugs, roflumilast and formoterol fumarate [99]. The MMAD of this formulation was 4.2–4.3 µm with an FPF greater than 50% of the emitted dose. In a mouse asthma model, the formulation decreased inflammation and remodeling, improving the mechanics of the lung [99].

Bahrainian et al. compared leucine and HP-β-CD for aerosolization capabilities in a spray-dried formulation of the antibiotic vancomycin [100]. The addition of HP-β-CD demonstrated greater potential than leucine in aerosolization, with an optimized formulation containing 17.39% HP-β-CD and 29.61% trehalose giving an FPF of 42.45 ± 1.22% [100]. Zhao et al. utilized HP-β-CD to develop a cyclodextrin-based drug carrier in combination with either lactose or raffinose [101,102]. In either case, the water uptake of the carrier was substantially reduced, with moisture uptake of 2–5% when HP-β-CD was included compared to 50–65% for lactose and raffinose alone [101,102]. When micronized budesonide was combined with the CD-based carriers through blending as a model drug, the FPF was improved by ~3.7-fold compared to spray-dried lactose or raffinose alone [101,102].

Spray–freeze drying was utilized to make inhalable particles of the model protein bovine serum albumin (BSA) with HP-β-CD as a stabilizer [103]. A design of experiment matrix was completed to identify parameters impacting performance. The greatest FPF was found to be 78–80% with an MMAD below 1 µm [103]. Of note, protein aggregation was found in all formulations. However, HP-β-CD prevented the fragmentation of BSA. The authors suggest the incorporation of HP-β-CD and an additional stabilizer to improve aggregation [103]. Vartiainen et al. utilized HP-β-CD as a stabilizer for DPIs of prednisone and fludrocortisone acetate via an aerosol flow reactor method [104]. The drugs demonstrated an FPF of 52–70% of the emitted dose and improved permeation [104]. Mohtar et al. compared variants of cyclodextrin: native β-CD, HP-β-CD, and sulfobutylether-β-cyclodextrin (SBE-β-CD) [105]. The three CD variants were formulated with fisetin via spray drying to improve drug solubility. SBE-β-CD demonstrated the greatest solubility enhancement. With the addition of 20 wt% leucine, the FPF was determined to be 75.83 ± 3.34% [105].

#### 5.3.4. Dextran

Dextran (Figure 5D) is a naturally occurring branched polymer that has been shown to elicit a wide range of therapeutic benefits. Inhaled dextran has been shown to prevent bacterial infections in mice by preventing pathogens such as *Pseudomonas aeruginosa* from binding to the epithelium of the respiratory tract [106]. Additionally, dextran has been shown to provide mucolytic activity in sputum from CF patients [107], as well as increase rates of mucociliary clearance in canine model systems [108]. Dextran has been gaining interest in recent years for incorporation within spray-dried DPI formulations.

Rifampicin particles were prepared as an aqueous suspension with dextran and spray-dried to yield porous MPs for inhalation [109]. At a ratio of 1:20 rifampicin–dextran 40, the FPF was 51.0 ± 1.28% [109]. Manser et al. spray-dried an AdHu5 labile adenoviral vector in a mannitol-dextran blend [110]. Incorporating higher molecular weight dextran at a greater mass ratio improved the retention of viral activity. The 1:3 ratio of mannitol to 500 kDa dextran minimized viral processing losses and exhibited the lowest MMAD of 4.4 µm. The emitted dose was 84% from an intratracheal dosator [110]. The authors note that greater dextran ratios may lead to moisture absorption and agglomeration [110]. As a result, this must be carefully considered and controlled.

**Figure 6:**
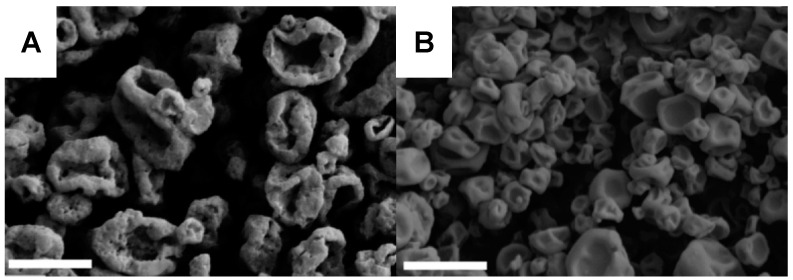
SEM micrographs of (**A**) curcumin-loaded acetylated dextran nanocomposite MPs and (**B**) curcumin-loaded acetylated dextran MPs. Scale bar = 2 µm. Figure reproduced with permission [112]. Copyright 2017, Elsevier.

Chemical modifications of dextran have also been utilized in DPI formulation studies. Shah et al. combined sulforhodamine B as a model water-soluble compound and acetylated dextran via spray drying [111]. The loading of sulforhodamine B was up to 16.7 µg/mg particle and demonstrated an MMAD of 2.06–2.86 µm with a >90% FPF. Sustained release of the model compound was found at pH 7, with faster release exhibited at pH 5 [111]. Acetylated dextran was also used by Wang et al. to deliver curcumin as a model drug (Figure 6) [112]. Two systems were evaluated: spray drying an aqueous NP suspension (Figure 6A) to prepare nano-in-MPs, and spray drying the components in organic solution to prepare MPs (Figure 6B). All systems were found to have an MMAD of around 2 µm (GSD of 2–3) [112].

Dextran was chemically modified to be amphiphilic and cationic using stearylamine for use in an antibiotic delivery formulation [113]. This lipopolymer was then formulated with the drug rifampicin through spray drying. The resulting particles could deaggregate into nanomicelles upon hydration [113]. Compared to unmodified dextran, the chemically modified analog led to a smaller MMAD and greater FPF. Where dextran alone had an MMAD of 4.77 µm and FPF of 62.98%, highly grafted dextran–stearylamine had an MMAD of 2.72 µm and FPF of 77.51% [113]. Waters and Hochhaus reported the preparation of dextran-budesonide conjugates to slow the release of the quickly absorbed drug [114]. The optimized formulation using a 40 kDa dextran-succinate-budesonide conjugate was spray-dried with lactose and exhibited an MMAD of 4.04 ± 1.42 µm (GSD = 3.97 ± 3.37) and FPF of 47.3 ± 17.9% [114].

#### 5.3.5. Hyaluronic Acid

Hyaluronic acid (HA; Figure 5E) is a glycosaminoglycan that is naturally found in the airway. Hyaluronic acid is a regulator of inflammation both in the airways and in other parts of the body [66,67,115]. It has been shown to increase mucociliary clearance rates in human cell cultures [116] and have no effect on inflammation in CF patients [117]. Other studies have shown that inhaled high molecular weight HA reduces inflammation in CF mice [118]. Recent studies have shown that inhaled HA decreases respiratory failure in COPD exacerbations [116]. In addition, HA, as part of the extracellular matrix, has been linked with preventing infections for a number of pathogens, including *Staphylococcus aureus*, *Haemophilus influenza*, and *Moraxella catarrhalis* [119]. As a result of these properties, multiple research groups have investigated the incorporation of HA in DPI formulations.

In 2003, Surendrakumar et al. co-spray-dried HA with insulin to create a powder with an MMAD between 1 and 4 µm [120]. The formulations were delivered to male beagle dogs and the presence of HA was found to extend the residence time of insulin in the plasma [120]. Li et al. co-spray-dried salbutamol sulfate with HA to form spherical, wrinkled MPs [121]. Without the need for a carrier, the FPF was greater than 30%. In vitro release of salbutamol sulfate lasted for 20 h, and pulmonary retention in a rat model was prolonged from 2 h to 8 h with reduced systemic exposure compared to spray-dried salbutamol sulfate alone [121]. The increased pulmonary retention was likely a result of the mucoadhesive properties of HA. Costabile et al. prepared co-spray-dried flucytosine with HA and mannitol using a one-step spray-drying process [122]. The optimal formulation had ~40% of the particles with an aerodynamic diameter less than 6.48 µm and ~25% less than 3.61 µm. It was noted in the study that the FPF at 90 L/min was 2-fold higher than the FPF measured at 60 L/min [122]. This may raise concerns in patients with decreased lung function. When delivered via intratracheal administration to rats, the HA-based formulation significantly increased drug levels in the bronchoalveolar lavage fluid and lung tissue compared to pure drug solution [122]. HA has also been used in spray-dried formulations with vancomycin. In this study, an ionic complex between HA (1–2 MDa) and vancomycin was prepared and then spray-dried as an aqueous dispersion [123]. Antibacterial efficacy was preserved against *Staphylococcus aureus* and methicillin-resistant *S. aureus*. The resulting particles exhibited an MMAD of 4.29 ± 0.03 µm and FPF of 42.9 ± 0.2% [123].

Liu et al. prepared budesonide submicron particles by wet ball milling and then subsequently loaded the drug into HA particles through spray drying [124]. The drug loading, MMAD, GSD, and FPF with respect to the emitted dose were reported as 21.0 ± 0.3%, 5.33 ± 0.05 µm, 1.68 ± 0.02, and 35.6 ± 3.3%, respectively [124]. As observed by others, the inhaled particles had a prolonged pharmacological effect in rats compared to the drug suspension [124], highlighting HA’s ability to promote increased retention. Fallacara et al. generated inhalable dry powders composed of urea-crosslinked HA and sodium ascorbyl phosphate, a molecule with anti-inflammatory properties [125]. Greater than 90% of the sodium ascorbyl phosphate was released within 4 h, and the respirable fraction was found to be 35.3 ± 0.3% [125]. In a later comparison, Nikjoo et al. prepared spray-dried MPs of HA (1.5–2.2 MDa) that had been crosslinked with urea or glutaraldehyde [126]. Crosslinking with glutaraldehyde gave the most favorable results, with FPF estimated to be 28 ± 2% [126]. A model drug was not incorporated within this system, so further studies must be conducted to evaluate the suitability of drug encapsulation and release.

#### 5.3.6. Cellulose Derivatives

Hydroxypropylmethylcellulose (HPMC) is a synthetically modified derivative of the natural biopolymer cellulose (Figure 5F). HPMC has FDA approval as a food additive and is considered safe for ingestion [127]. A report from 1986 noted that HPMC was practically nontoxic when administered through the inhalation route [128]. The use of HPMC capsules is becoming increasingly common for dry powder delivery in capsule-based DPI devices as an alternative to hard gelatin capsules. Multiple commercial products are now available that use HPMC capsules [23]. A few studies have evaluated the use of HPMC as an excipient within the dry powder formulation itself. In 2011, Shah et al. reported a co-spray-dried formulation that contained the drugs salmeterol xinafoate and fluticasone propionate with the excipients leucine and HPMC [129]. In this work, HPMC was added to control the drug release profile. Indeed, increasing the HPMC content slowed the release of the drug [129]. Unfortunately, aerodynamic characterization was not included in this work, so the suitability of the particle system as a formulation for DPI delivery cannot be adequately assessed.

Leslie et al. reported the use of HPMC in developing an inhaled drug of nitrofurantoin [130]. In this study, spray drying with a two-fluid nozzle was compared to a three-fluid nozzle. For the two-fluid nozzle, HPMC and nitrofurantoin were mixed into a single solution and spray-dried. For the three-fluid nozzle, HPMC and nitrofurantoin solutions were spray-dried separately so that the drug was encapsulated in a layer of HPMC. In almost all aspects, the two-fluid nozzle-generated powders demonstrated superior performance. For example, the powders prepared with the two-fluid nozzle demonstrated an MMAD of 1.78 ± 0.01 µm and FPF of 87.4 ± 2.8% whereas the powders prepared with the three-fluid nozzle demonstrated an MMAD of 4.36 ± 0.4 µm and FPF of 40.0 ± 5.8% [130]. From this data, it can be interpreted that aerodynamic performance was improved when the HPMC was interspersed throughout the particle rather than predominantly at the particle surface.

Carboxymethylcellulose (CMC) is a linear, semi-synthetic polymer derived from the natural biopolymer cellulose (Figure 5F). CMC is frequently utilized in the food and pharmaceutical industries but has recently demonstrated pro-inflammatory effects on the gut epithelia [131]. The presence of CMC in the literature for DPI applications is limited. In 2017, Gallo et al. compared three polysaccharides to improve the drug residence time of sodium cromoglycate in the lungs [132]. The use of CMC, HA, and alginate was compared. Similar results were seen for the three biopolymers, but the powder with CMC exhibited the greatest mucoadhesion and FPF. For CMC, HA, and alginate, the MMADs were 4.09 ± 0.23 µm, 4.21 ± 0.15 µm, and 4.16 ± 0.19 µm, and the FPFs were 37.62 ± 2.75%, 28.53 ± 4.17%, and 31.05 ± 1.97%, respectively [132].

#### 5.3.7. Combination Systems

**Figure 7:**
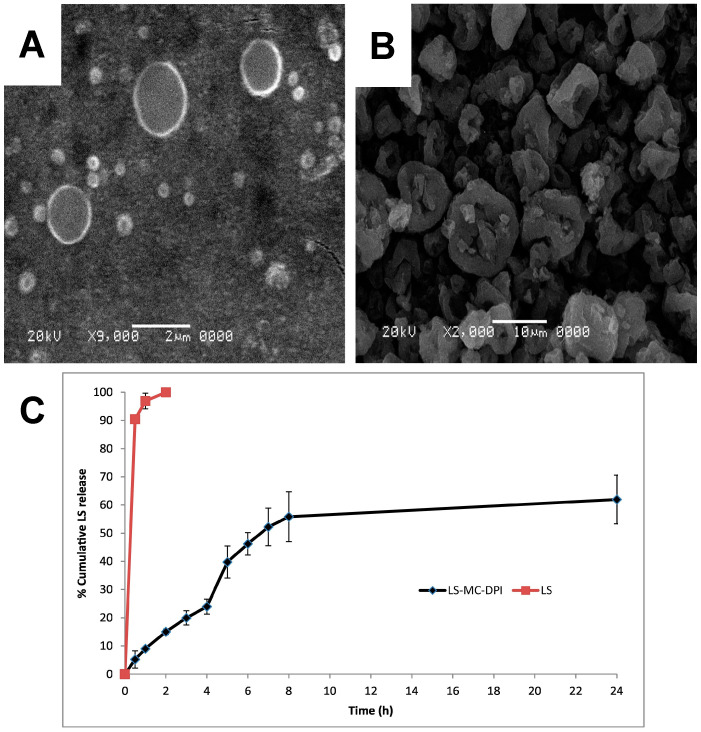
SEM photomicrographs of (**A**) losartan microplex at 9000× magnification (scale bar = 2 µm) and (**B**) spray-dried losartan microplex at 4000× magnification (scale bar = 10 µm). (**C**) In vitro release profile of losartan (LS, red square) and spray-dried losartan microplex (LS-MC-DPI; black diamond) over 24 h. Figure reproduced with permission [133]. Copyright 2019, Elsevier.

Multiple research groups have investigated combinations of native or native-derived biopolymers. Elmowafy and Soliman prepared a system that incorporated both chitosan and dextran [133]. Briefly, losartan was encapsulated within a self-assembled chitosan and dextran sulfate microplex (Figure 7A), which was then spray-dried (Figure 7B). Dextran was specifically included in this case as a drying adjuvant, and the resulting product had a low moisture content of 2.16% [133]. The release of the drug was extended to more than 24 h (Figure 7C), and the respirable fraction was reported to be 74.00 ± 0.25% [133]. Chitosan, a net-positive polysaccharide, has also been combined in DPI systems with HA and alginate, net-negative polysaccharides [134,135,136,137]. Isoniazid was loaded within a complex of HA and either chitosan, thiolated chitosan, or mannosylated chitosan. Thiolated and mannosylated chitosan were evaluated to improve mucoadhesion and target mannose receptors in alveolar macrophages, respectively [134]. Furthermore, this report compared freeze-drying and spray-drying methods. Overall, the spray-dried samples had superior flowability and morphology, but the aerodynamic properties were similar between the two preparation methods [134]. Freeze-dried and spray-dried particles exhibited MMADs of 2.67–2.981 µm and 1.632–4.132 µm, respectively [134].

Putri et al. combined chitosan and alginate with rifampicin via spray drying [135]. The optimal candidate had a composition of 2:1:1 rifampicin–chitosan–alginate. Drug release in simulated wound fluid and simulated macrophage fluid was 78.301 ± 1.332% and 41.355 ± 1.259% in 2 h, respectively [135]. The greatest aerodynamic performance included an MMAD of 11.4288 ± 1.259 µm [135]. Given that particles of 1–5 µm aerodynamic size have the greatest chance of reaching and depositing within the lungs, further work will be needed to reduce the size of this system and improve the potential for efficient inhaled delivery. Two studies have been reported that combined alginate and chitosan with budesonide [136,137]. Budesonide particles were prepared through controlled gelation of alginate, calcium chloride, and chitosan followed by lyophilization with mannitol [136,137]. The MMAD, GSD, and FPF of the product was 1.16 ± 0.01 µm, 3.78 ± 0.07, and 56.18 ± 0.05%, respectively [136,137]. Controlled release of the drug was observed over 24 h in vitro [136,137], and a 14-fold increase in lung deposition was reported in rats [136].

Studies have also combined alginate with β-CD. Patil et al. spray-dried alginate MPs containing both rifampicin and β-CD [138]. The drug content was between 62 and 80%, and the optimized formulation had an MMAD, GSD, and FPF of 5.4 µm, 1.8, and 39.5%, respectively [138]. Using this system, rifampicin was detected in the plasma of rats from 4 h to 72 h, whereas the free drug was cleared within 24 h [138]. Mahmoud et al. prepared an emulsion with multiple components, including alginate, isopropyl myristate (oil phase), Tween 80 (surfactant), calcium β-glycerophosphate (crosslinking agent), and roflumilast (drug), which was then spray-dried [139]. The FPF for pure drug was 9.7 ± 4.4%, which was increased to 36.4 ± 2.2% with the emulsion-based formulation [139]. However, adding multiple excipients in great quantities reduces the drug content substantially compared to spray drying pure drug, as the drug loading ranged from 4.02 ± 0.26 to 5.62 ± 0.25% in the emulsion-based formulation [139], raising the question of whether the improvement in FPF leads to a greater delivered dose of the active ingredient.

## 6. Synthetic Biopolymers in DPI Formulations

### 6.1. Synthetic Biopolymer Overview

Synthetic biopolymers are defined in various ways throughout the literature. For the purposes of this review, we will focus on laboratory-synthesized biopolymers, including those synthesized from bio-derived or synthetic monomers, that follow natural degradation paths in vivo [140,141]. A range of synthetic biopolymers has been investigated for use as carriers and excipients in DPI formulations (Figure 6). These synthetic biopolymers have been approved by the FDA for one or more specific biomedical applications (e.g., implant, ointment, oral formulation) and/or as a food additive (e.g., emulsifier, stabilizer); however, no synthetic biopolymer has yet been approved for inhalation. While not approved in commercial inhalation products at this time, the use of synthetic biopolymers in dry powder formulations is increasing in popularity throughout the research community.

### 6.2. Synthetic Biopolymers in DPIs

#### 6.2.1. Polycaprolactone

**Figure 8:**
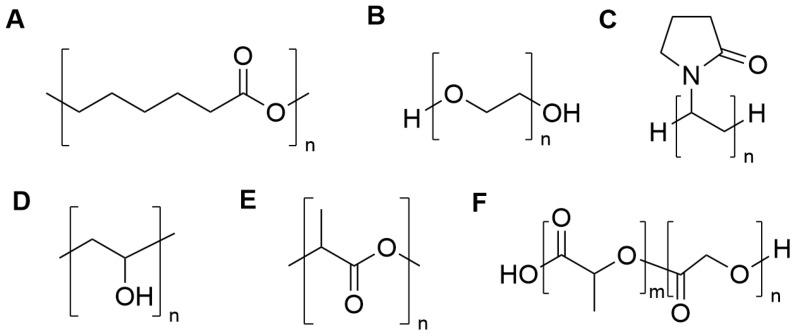
Chemical structures of synthetic biopolymers, including (**A**) polycaprolactone, (**B**) poly(ethylene glycol), (**C**) poly(vinyl pyrrolidone), (**D**) poly(vinyl alcohol), (**E**) polylactic acid, and (**F**) poly(lactic-co-glycolic acid).

Polycaprolactone (PCL; Figure 8A) is a synthetic, hydrophobic, biodegradable polymer that is frequently used in tissue engineering applications [142]. Over the past 15 years, PCL has garnered interest for use in inhaled drug delivery applications. Tuli and coworkers prepared PCL MPs as an alternative carrier to coarse lactose [143,144]. Due to the high potency of salbutamol sulfate, blending the drug at 2.5% *w*/*w* was sufficient for efficacy. Here, PCL (80 kDa) MPs (26, 48, 100, and 150 µm) were prepared using the oil-in-water solvent evaporation method and then dry coated with salbutamol sulfate. The authors discovered that coating the PCL MPs with either magnesium stearate or leucine prior to coating with the drug increased the FPF metrics to 11–20% [143,144].

**Figure 9:**
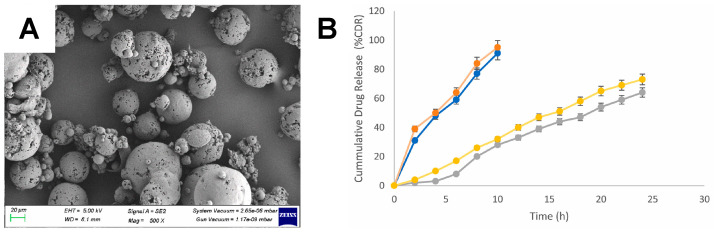
(**A**) SEM image of optimized linezolid-loaded PCL microspheres at 500× magnification. Scale bar = 20 µm. (**B**) Drug release of free linezolid in phosphate buffer with a pH of 7.4 (blue) and acetate buffer with a pH of 4.4 (orange) compared to drug release from linezolid-loaded PCL microspheres with pHs of 7.4 (gray) and 4.4 (yellow). Figure reproduced with permission [146]. Copyright 2023, Elsevier.

In contrast to high-potency drugs that are frequently employed for the treatment of asthma and COPD, pulmonary delivery of antibiotics requires a greater dose to elicit a response. In these cases, the MPs are often engineered to reach the lungs without a carrier particle. Kho et al. reported the preparation of ~270 nm PCL (80 kDa) NPs that encapsulated levofloxacin [145]. The NPs were then spray-dried to formulate NP aggregates with calculated aerodynamic diameters in the range of 3 to 4 µm. The addition of lactose and leucine excipients assisted with the return of the aggregates to the primary NPs upon dissolution and flowability, respectively [145]. The authors reported that the return to primary NPs following lung deposition is desired as the small NPs may permeate through the mucus mesh and exhibit longer retention times in the lungs [145]. However, levofloxacin was encapsulated at a relatively low drug loading of 0.4% *w*/*w*, limiting the potential for high-dose antibiotic delivery [145]. Sharma et al. fabricated linezolid-loaded PCL (80 kDa) MPs using a double emulsion solvent evaporation method with drug loading of up to 22% (Figure 9A) [146]. The release of linezolid was sustained over 24 h at both pH 7.4 and pH 4.4 in contrast to the rapid release of pure linezolid (Figure 9B). Furthermore, the PCL MPs exhibited an FPF of 62.56 ± 0.89%, a substantial improvement over pure linezolid (FPF of 42.69 ± 1.27%) [146]. Parikh and coworkers prepared isoniazid-loaded PCL MPs (64.83% *w*/*w* isoniazid loading) through a double emulsification method followed by spray drying [147]. The PCL MPs exhibited a similar FPF to MPs prepared for isoniazid alone (51.83% and 54.85%, respectively). However, the PCL MPs exhibited faster drug release in alveolar lung fluid (pH 4.5) compared to pH 7.4, an opposite result from that observed with pure isoniazid MPs, highlighting the potential for the PCL-based particles to deliver the drug more effectively to alveolar macrophages [147]. Kasten et al. prepared low-density, azithromycin-loaded PCL (10 kDa) MPs using the water-in-oil-in-water double emulsion method with azithromycin content ranging from 3.50 to 23.07% *w*/*w* [148]. Calculated aerodynamic diameters ranged from 2.29 to 8.99 µm, and in vitro drug release at pH 7.4 was maintained over 24 h [148].

Topal and coworkers prepared MP composites of ciprofloxacin-loaded PCL NPs [149]. Briefly, NPs were prepared using a solid-in-oil-in-water emulsion solvent method, dispersed in mannitol solution, and freeze-dried. As mannitol readily dissolves in an aqueous solution, the authors rationalized that the composing NPs would be readily released upon dissolution within the lungs. The addition of a chitosan coating on the PCL NPs increased the encapsulation efficiency from 2.95 ± 0.01 to 8.26 ± 0.02% and reduced the rate of drug release [149]. The optimized formulation exhibited an MMAD of 5.4 µm and a modest FPF of 20.23% [149]. Further reduction in the MMAD would likely facilitate a greater FPF.

#### 6.2.2. Poly(ethylene glycol)

Poly(ethylene glycol) (PEG; Figure 8B) is a water-soluble synthetic biopolymer made of repeating ethylene glycol units. PEGs are commonly found in various drug delivery systems, such as providing the base in ointments [150]. A vast range of PEG molecular weights is commercially available, and the molecular weight controls its potential for biodegradation in the body [150]. PEGylation, or the attachment of PEG moieties to molecules and macrostructures, is frequently utilized in many drug delivery applications. PEGylation of phospholipid-based nanocarriers for inhalation has been widely examined and is summarized in a thorough review by Muralidharan et al. [151]. As such, the PEGylation of such carriers will not be discussed in this review and the focus will remain on studies where PEG was used in a co-formulated dry powder.

Corrigan et al. reported the co-spray drying of salbutamol sulfate and either PEG4000 or PEG20000 [152]. The focus of this work was to elucidate the impact of adding PEG at 5, 20, and 40 wt% on the crystallinity of the final product. Amorphous materials are typically achieved with spray-drying techniques, and this is frequently seen to be beneficial in improving the bioavailability of the resulting drug formulation [8]. In the co-formulation of salbutamol sulfate with PEG4000, it was found that only the 5 wt% ratio exhibited no X-ray diffraction peaks to indicate crystallinity. For PEG20000, 5 and 20 wt% were both found to be amorphous [152]. However, the authors postulated that such systems may not be stable for room temperature storage due to the low glass transition temperatures of PEGs [152]. Room temperature storage is preferred for DPI products to minimize costs associated with cold-chain storage and improve patient accessibility [153]. Gilani and coworkers compared the delivery of beclomethasone dipropionate blended with spray-dried lactose and with lactose co-spray-dried with PEG400, PEG3000, or PEG6000 [154]. The authors found that the addition of PEG improved the fluidization capabilities of the powder, increasing the emitted dose from 29 ± 1.5% with spray-dried lactose to 82–92% with the three PEGs. Furthermore, the use of PEG3000 or PEG6000 improved the FPF to 24–26% compared to the 14–15% FPF achieved with either spray-dried lactose or the PEG400 co-sprayed formulation [154].

#### 6.2.3. Poly(vinyl alcohol) and Poly(vinyl pyrrolidone)

Poly(vinyl pyrrolidone) (PVP; Figure 8C) and poly(vinyl alcohol) (PVA; Figure 8D) are synthetic, water-soluble, and biodegradable polymers [155]. These synthetic vinyl polymers are actively used in a wide range of biomedical applications, including in oral drug formulations, and are FDA-approved excipients [156,157]. In 2008, Buttini et al. coated budesonide MPs with PVA or PVP by adsorption followed by spray drying [158]. The drug particles were then blended with lactose and loaded into a DPI device. Coating the budesonide with the vinyl biopolymers increased the FPF from 29.1 ± 0.7% to 52.8 ± 1.0% using 0.01% PVA or 52.3 ± 1.5% using 0.01% PVP [158]. The authors describe the vinyl polymers as modifying the adhesive interactions between MPs and postulate that these biopolymers could function as an alternative to leucine in formulations for improving dispersion [158].

Inhalable ciprofloxacin-loaded PVA MPs were also prepared by spray drying, with a drug encapsulation of >90% and drug content up to 10 wt% [159]. Ciprofloxacin sustained release was observed over 24 h, and the particles demonstrated potential for inhalation with an MMAD of 5.06 ± 0.10 µm and FPF of 39.78 ± 0.98% [159]. In an alternate method, Party et al. prepared meloxicam and PVA NPs via wet milling and then spray-dried the particles with or without leucine to form nano-in-micro particles [160]. The NPs (120–140 nm) could be visualized within the MPs using scanning electron microscopy. The NPs improved the dissolution of poorly water-soluble meloxicam, with potential explanations of a nanosizing effect, higher specific surface area, or amorphization [160]. The FPF of the MPs, with or without leucine, was 72–76%, indicating a high potential for lung deposition [160].

#### 6.2.4. Polylactic Acid

Polylactic acid (PLA; Figure 8E) is a biodegradable, hydrophobic, synthetic polymer prepared from naturally occurring lactic acid monomers [161]. PLA has been approved by the FDA for use in various applications, including those involving direct contact of the material with biological fluids [162,163]. In 1998, El-Baseir et al. developed PLA microspheres containing either nedocromil sodium or beclomethasone dipropionate [164]. Extended drug release was found, with release for 6 d and 8 d for beclomethasone dipropionate and nedocromil sodium, respectively. For the beclomethasone dipropionate particles, approximately 42% had an aerodynamic diameter of less than 5 µm [164]. PLA MPs were also prepared using precipitation with compressed antisolvent (PCA) technique, with supercritical CO_2_ as the antisolvent [165]. This technique did not include the use of any model drugs, but the authors hypothesized that a drug could be incorporated within the PLA powder [165]. However, evaluation using a model throat cast of a human adult male indicated that the majority of the powder deposited in the throat and not in the lungs when released from a Turbuhaler at 28.3, 60, and 90 L/min. In all cases, less than 20% was estimated to reach the lungs [165]. Further optimization of PLA MPs is needed to improve their potential for DPI use.

#### 6.2.5. Poly(lactic-co-glycolic acid)

Poly(lactic-co-glycolic acid) (PLGA; Figure 8F) is a copolymer of PLA and polyglycolic acid (PGA) [166]. This synthetic polymer spans a range of molecular weights and ratios of PLA to PGA residues, with such properties controlling drug release and polymer degradation. A thorough description of PLGA as a biodegradable drug carrier has been previously published by Makadia and Siegel [166]. From a regulatory perspective, the FDA has approved over 10 sustained-release products based on PLGA MPs [167]. While there is a wide variety of DPI formulations based on PLGA in the literature, to date, no PLGA-based systems have been approved for inhalation applications.

Diab et al. reported on the preparation of PLGA NPs to deliver rifampicin, a first-line tuberculosis drug [168]. The NPs were prepared using an oil-in-water emulsion solvent-evaporation method and then lyophilized. Rifampicin loading of the NPs ranged from 7.0 ± 0.2% to 42.4 ± 2.2% *w*/*w*, and 71% of the drug was released over 1 week [168]. With an MMAD of 4.5 µm and FPF of 52%, the authors report that inefficient deaggregation was observed during aerosolization [168]. Using a similar methodology, Vanza et al. reported on the preparation of PLGA NPs loaded with the anticancer agent afatnib [169]. During the lyophilization step, a cryoprotectant, trehalose trihydrate, was added at a 1:2 PLGA–trehalose ratio. Drug release was sustained compared to a plain drug, with 80% of the drug released at 18 h, 24 h, and 34 h at pH 5.5, 6.5, and 7.4, respectively [169]. The MMAD was smaller than Diab et al. observed for rifampicin at 2.79 ± 0.01 µm (FPF of 63.29 ± 0.11%) [168,169].

**Figure 10:**
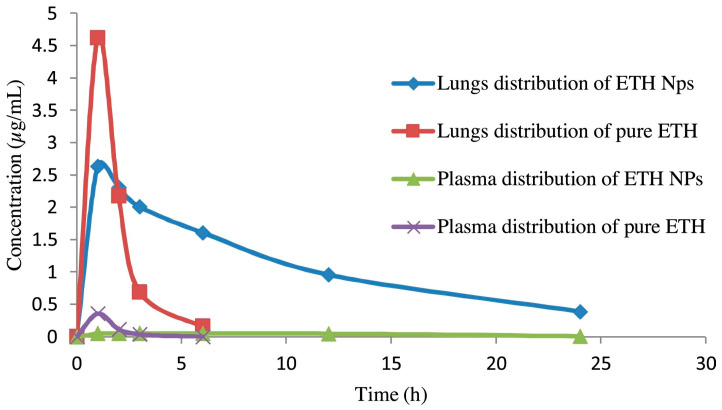
Lung and plasma distribution of ethionamide (ETH) in NP DPI formulation (ETH NPs) or as pure drug. Figure reproduced with permission [170]. Copyright 2017, Elsevier.

Debnath et al. prepared ethionamide-loaded PLGA NPs using a similar emulsion solvent evaporation method and lyophilization with mannitol as the cryoprotectant [170]. However, these particles were then mixed manually with a coarse lactose carrier for DPI use to improve delivery. The PLGA NPs were 225.7 ± 4.56 nm but with the lactose carrier demonstrated an MMAD of 1.79 µm and FPF of 94.38%. More than 95% of the drug was released within 24 h in vitro in simulated lung fluid [170]. Upon pulmonary delivery of the pure ethionamide drug or the DPI formulation containing ethionamide-loaded PLGA NPs to rats, it was found that the NP-based formulation localized delivery to and increased drug residence time in the lungs (Figure 10) [170]. Similar methods have been reported to prepare PLGA particles for inhalation loaded with biologics. In 2020, Sato et al. reported PLGA-based MPs that contained salmon calcitonin, a model peptide [171]. Fine droplet drying (FDD) was used to prepare the MPs, leading to a high loading efficiency and minimal peptide degradation. The peptide was released over 24 h in vitro in simulated lung fluid. Upon mixing with a coarse lactose carrier for DPI delivery, the reported MMAD was 4.1 µm and FPF was 28% [171].

As was seen with other polymers, spray drying is also frequently employed for developing inhaled formulations of PLGA. Two major approaches are used: spray drying PLGA NP/MP suspensions after formation or spray drying the precursor PLGA solution. Scharließ et al. reported the preparation of ovalbumin-loaded PLGA nano-in-MPs [172]. A double emulsion solvent evaporation method was utilized to first generate 270 nm PLGA NPs with up to 10% ovalbumin, which was then spray-dried into a respirable powder with trehalose. The NPs were redispersable upon dissolution, and the FPF was approximately 50% of the loaded dose [172]. Varshosaz et al. also reported on PLGA NPs (240.4 ± 19.5 nm) that were formed and then spray-dried, with the NPs encapsulating tadalafil [173]. Co-spray drying the NP suspension with inert excipients, including mannitol, leucine, and lactose, yielded MPs with an MMAD of 1.4 to 2.8 µm, with those spray-dried with lactose, lactose and leucine, or mannitol and leucine exhibiting FPFs between 50 and 60% [173]. In 2021, Yurdasiper et al. prepared PLGA MPs via an emulsion solvent evaporation method followed by spray drying with mannitol and leucine [174]. The MMAD ranged from 2.5 to 4.1 µm with a respirable fraction of 70–79% [174].

Ohashi et al. and Tavares et al. reported different approaches to spray drying, where a pre-formulation step of the PLGA NPs was not included [175,176]. Using a four-fluid nozzle, rifampicin and PLGA NPs were spray-dried, with rifampicin and PLGA in an acetone/methanol solution and mannitol in an aqueous solution [176]. The resulting NPs were 213 nm and were retained following the dissolution of the MPs in water [176]. While aerodynamic testing was performed, the MMAD and FPF were unfortunately not reported. Using an alternative method, PLGA MPs containing bovine serum albumin (BSA), a model protein, were spray-dried with leucine via supercritical CO_2_-assisted spray drying [175]. The MPs that combined PLGA, BSA, and leucine exhibited an MMAD of 2.9 ± 0.8 µm and FPF of 43.4 ± 3.7% [175].

Lastly, PLGA large porous particles also represent an approach to a PLGA-based DPI formulation. Shiehzadeh et al. reported on the preparation of PLGA large porous particles loaded with gentamicin sulfate [177]. The particles were prepared using a double emulsion (*w*/*o*/*w*) solvent evaporation method including ammonium bicarbonate as a pore-forming agent. The porous structure facilitated a fast and near complete drug release (>93% release in 30 min). While the particles exhibited a large geometric diameter (>10 µm), the highly porous nature of the particles led to an MMAD of 4.98 ± 0.1 µm and FPF of 39.07 ± 1.5% [177]. Zhu et al. also used a double emulsion (*w*/*o*/*w*) solvent evaporation method with ammonium bicarbonate to prepare PLGA large porous particles loaded with oridonin, resulting in particles with a geometric diameter of 11.6 ± 2.3 µm [178]. The MMAD was reduced to 2.7 ± 0.3 µm due to the low density of the porous particles, giving an FPF of 29.65% [178]. Importantly, 74% of the drug was released within 1 h, and uptake by macrophages did not occur until after 8 h. The authors postulate that the system could deliver the drug prior to phagocytosis [178].

## 7. Considerations for Biopolymeric Systems in Inhalation Applications

### 7.1. Polymeric Attributes

The molecular weight of biopolymers can be controlled to alter the properties of the resulting nano- or microparticles. The molecular weight can control solubility, degradation kinetics, drug release properties, and other physiological responses. For example, HA exhibits contrasting effects in its high and low molecular weights. HA that is >1 MDa is often considered to be high molecular weight and exhibits immunosuppressive and anti-inflammatory effects [66]. Upon degradation to lower molecular weights (1–800 kDa), HA signals for immunostimulatory, angiogenic, and pro-inflammatory activity [66]. Both size regimes have benefits for differing applications, allowing for a single biopolymer to have multifaceted roles. The polymeric molecular weight must be considered when choosing a biopolymer for the development of a DPI formulation. Furthermore, the molecular weight must be aligned with the desired penetration through the mucus and periciliary layer [30].

Many of the biopolymers described herein have the potential for modification to alter physicochemical (e.g., solubility, charge) and targeting properties. The polysaccharides in Figure 5 have functional groups that allow for chemical modification, such as primary alcohols, primary amines, and carboxylic acids. For example, β-CD is frequently chemically modified to form HP-β-CD to improve its water solubility and reduce toxicity [179]. Chitosan, a net-positive polysaccharide, has been mannosylated to target alveolar macrophages and thiolated to improve mucoadhesion [134]. However, careful considerations must be made to ensure that changing the structure of such biopolymers does not lead to toxicity concerns or a reduction in biodegradation potential.

Some polymers have exhibited antibacterial properties in reported studies, allowing their use in DPI formulations to serve as an additional active component rather than an inert excipient. Chitosan has been reported to exert action on the cell surface of bacteria through electrostatic interactions, leading to permeabilization of the bacterial cell. As noted in a comprehensive review by Li et al., the antibacterial efficacy of chitosan can be influenced by the molecular weight, chitosan source, and degree of deacetylation, among many other factors [180].

Biopolymeric NPs and MPs can have prescribed dissolution kinetics, which can be advantageous for time-released formulations. Time-release may be preferable for therapies designed to alter mucus itself as a particle designed to land in an airway of the lung with mucus plugging or a stagnant mucus flow. A time-release formulation that cleaves the disulfide bonds that stitch mucin monomers into long-chain polymers [32,42] may prolong the documented decreases in mucus viscoelasticity and increase transport rates [41,42,44,181]. The incorporation of such mucolytic biopolymers in DPI formulations may allow for improved efficacy of antibiotics or other drugs and biologics, as permeation of the active ingredient through the ASL may be improved. In the case of muco-obstructive pulmonary diseases, where the mucus is hyperconcentrated and the pores in the mucus mesh are smaller, the need for enhanced diffusion through the ASL becomes even more essential.

Some applications have worked to design biopolymers that are mucoadhesive or adhesive to the airway epithelium. When designing a particle that strongly interacts with the ASL, particles must be engineered to avoid forming crosslinks between constituents of the mucus layer, as was demonstrated with chitosan [64]. Additionally, particle design should avoid increasing the adhesion of the mucus layer to the underlying epithelium and the cohesion of the mucus layer with itself, with both having been shown to decrease cough and mucociliary clearance [182].

### 7.2. Safety Considerations

All of the biopolymers reported herein exhibit some level of biodegradation in certain forms. However, this is not the case for every variety of biopolymers, necessitating careful consideration when selecting the polymer and its attributes (e.g., molecular weight, functionalization). Biodegradation can occur via enzymatic degradation, hydrolysis, or a mixture of both [141]. For example, the biopolymers HA and chitosan are degraded in vivo via enzymatic processes [141]. The enzyme hyaluronidase is native to humans and rapidly catalyzes the breakdown of HA [66], whereas lysozyme is the primary mechanism of degradation for chitosan in humans [183]. Many biopolymers, including PLGA, PLA, PCL, PVA, and PVP, degrade in vivo through hydrolytic methods [141].

For many biopolymers, degradation and/or clearance are highly influenced by the molecular weight. It has previously been reported that alginate should be below 50 kDa for renal clearance as mammals do not have the enzymes required to degrade alginate (i.e., alginase) [184]. Partially oxidized alginate chains, however, can degrade in aqueous solution [185]. The molecular weight of chitosan dictates its biodegradation. Furthermore, chitosan with high degrees of deacetylation has been noted to have slower degradation than those with lower degrees of deacetylation [183]. While PLA is known to degrade through hydrolysis, the degradation of PLA depends on the molecular weight and the chirality of the monomers [186]. PCL is known to undergo hydrolytic cleavage of ester linkages, but studies have shown that PCL at a low molecular weight (<3000 Da) can undergo intracellular degradation [148]. These examples are not an exhaustive list but instead highlight the need for consideration of biodegradation process when selecting a biopolymer for use in pulmonary applications.

Further attention must be given to the duration of the biodegradation processes. While these biopolymers are considered biodegradable, they may not be degraded quickly enough to prevent bioaccumulation in the lungs upon repeated use. Reddy et al. reported a review of in vitro biopolymer degradation studies mainly established for tissue engineering applications, with some polymers degrading as quickly as 1–4 weeks and others seeing incomplete degradation after 24 weeks [141]. The small, low-density nature of the NPs and MPs may lead to faster degradation than was reported in this review; however, the effect of daily, or more frequent, dosing must be thoroughly considered. A comprehensive and systemic toxicity study of inhaled biopolymers has not yet been reported and is a necessary step in moving forward with biopolymer use in DPI formulations.

### 7.3. Stability upon Storage

DPI formulations are often amorphous due to processing conditions, notably spray drying. While being amorphous is beneficial in that it may improve the bioavailability of the drug compared to a crystalline counterpart [8], stability becomes a concern. When storing a dry powder formulation, low-humidity environments are preferred to prevent water uptake. Furthermore, the amorphous material is typically regarded as stable for storage if the storage temperature is 40–50 °C less than the glass transition temperature [187]. Corrigan et al. noted that PEGs have very low glass transition temperatures, indicating that storage at room temperature may not lead to aerodynamic stability for DPI formulations prepared containing great amounts of PEG [152]. The glass transition temperature of each polymer should be considered when preparing a DPI formulation.

### 7.4. Manufacturing

While the use of biopolymeric excipients for DPIs mostly remains in the research stage at this time, considerations toward manufacturing and industrialization should be examined. In order to produce biopolymeric DPI formulations on a scale suitable for commercialization, production-scale manufacturing is required. Successful scale-up from bench-top methods to pilot and production scale will be imperative. Furthermore, considerations need to include standardized methods to establish quality in order to meet regulatory requirements. All reagents would be subject to standard compendial requirements for the absence of impurities, foreign particulates, and microbiological contamination. With respect to quality, analytical methods to establish the molecular weight and dispersity of polymers would be required, utilizing techniques such as intrinsic viscosity measurements and Mark–Houwink parameters, gel permeation chromatography, or light scattering [188,189]. Careful attention should be given to choosing the appropriate technique for each specific system. In addition, the stability of the product would need to be established following standard guidelines [28,190,191].

There are relevant compendial standards that would be suitable for characterizing the quality of the product at the commercial stage, including but not limited to USP General Chapters <1153> (drug products containing nanomaterials) [192], <5> (inhalation and nasal drug products) [193], <601> (performance quality tests for inhalation and nasal drug products) [27], <1> (injections and implanted drug products) [194], and <88> (biological reactivity to polymeric materials) [195]. Importantly, the regulatory agencies are currently working to expand statutes in this research area, with USP soliciting input on the addition of General Chapter <1156> in 2024, a chapter focused on product quality and performance tests for microsphere drug products. The addition of this chapter will provide needed guidance in developing polymeric microparticle drug products.

Multiple products using polymeric microparticle systems have been approved by the FDA, albeit not for inhalation. The long-acting injectable microparticle PLGA formulations Lupron Depot^®^ and Risperdal Consta^®^ are two examples of this technology [196,197]. The fact that such products exist suggests that manufacturing biopolymeric DPI formulations would be a technical challenge but not an insurmountable barrier.

## 8. Conclusions and Future Insight

While no biopolymeric excipient has been approved at this time for inhalation applications, there is a wide library of research studies that have investigated this path forward. At this stage, many limitations exist in that the current studies reported are primarily in vitro analyses. Toxicity testing upon pulmonary delivery of these biopolymers is lacking and will need to be more thoroughly investigated prior to moving these systems forward.

As was demonstrated in this review, the dosage of the drug required to reach the target area dictates what formulation elements are possible. For drugs like antibiotics where large quantities are needed to reach the lungs, the incorporation of great amounts of biopolymeric excipients may not be advised, as the resulting drug content of the powder will necessitate doses that are not feasible. However, the use of biopolymers that provide beneficial attributes, such as the antibacterial effects of chitosan, mucolytic activity of low molecular weight alginate, or increased lung retention of HA, may prove useful in reducing the required dose of the active pharmaceutical ingredient. Controlled release and targeted delivery represent avenues that may also aid in reducing the required dose or frequency of dose, ultimately improving patient compliance and satisfaction.

## Figures and Tables

**Table 1:** Mucus, mucins, and DNA concentration for healthy subjects, as well as patients with non-cystic fibrosis bronchiectasis (NCFB), and CF.

Disease State	[Mucus] (% os)	[Mucins] (mg/mL)	[DNA] (mg/mL)
Healthy [36]	1.1	1.8	0.009
NCFB [36]	1.8	4.6	0.280
Adult CF [37,39,40]	4.9	10.8	2.1

## Data Availability

Not applicable.
